# Supplementary material for: Variants of beta-glucan polysaccharides downregulate autoimmune inflammation
Source: Commun Biol. 2022 May 12;5:449. doi: 10.1038/s42003-022-03376-y (PMC9098905; doi:10.1038/s42003-022-03376-y)
Supplement: Supplementary file 2 — Supplementary Information [file 42003_2022_3376_MOESM2_ESM.pdf]

Variants of beta-glucan polysaccharides downregulate autoimmune inflammation

Cecilia Fahlquist-Hagert[1], Outi Sareila[1,2], Sofia Rosendahl[2], Rikard Holmdahl[1,2]

1. Medical Inflammation Research, MediCity Research Laboratory, University of Turku, FI-20520 Turku, Finland.
2. Medical Inflammation Research, Department of Biochemistry and Biophysics, Karolinska Institute, SE-17177 Stockholm, Sweden.

Current affiliation of Cecilia Fahlquist-Hagert is Aarhus University, Department of Biomedicine, C. F. Møllers Allé 6, 8000 Aarhus, Denmark

Current affiliation of Outi Sareila is Department of Rheumatology and Inflammation Research, Institute of Medicine, Sahlgrenska Academy, University of Gothenburg, 40530 Gothenburg, Sweden.

Corresponding authors: Cecilia Fahlquist-Hagert, cecilia.hagert@biomed.au.dk, +46-703879996 and Rikard Holmdahl, rikard.holmdahl@ki.se, +46-(0)8-524 846 07

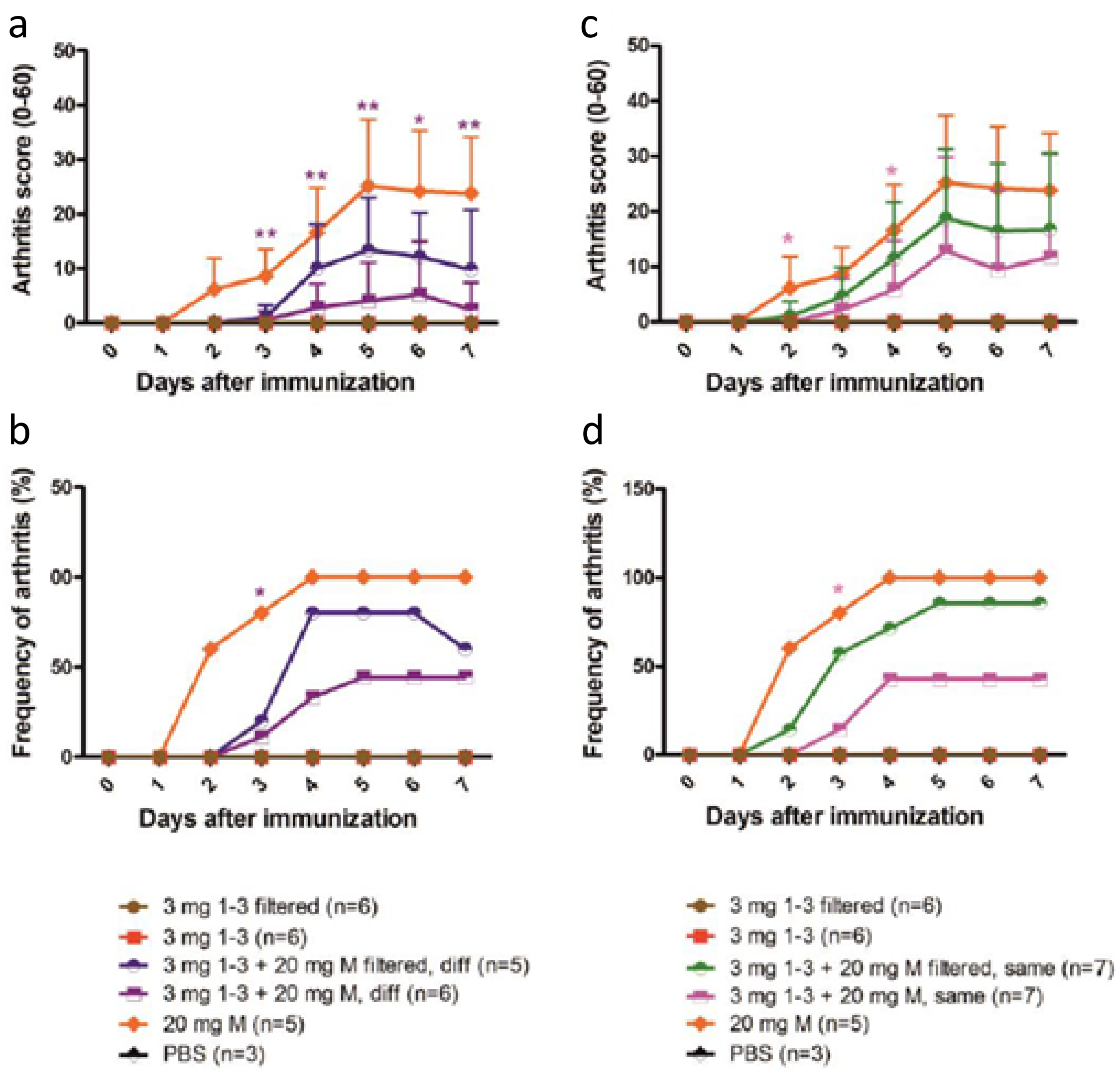

Supplementary Figure 1. Comparison of filtered and unfiltered glucan. a-b) A suspension of curdlan (1,3-β-glucan) used unfiltered (oranges squares) and as filtered (brown circle) cannot induce arthritis. In parallel mice were treated with mannan alone (orange diamond) or in combination with filtered (blue half-filled triangles) or unfiltered curdlan (purple half-filled squares). Mannan and curdlan was injected at different sides of the peritoneum. c-d) As a comparison (in parallel with a-b), non-filtered (light purple half-filled square) and filtered curdlan (green half-filled circle) in combination with mannan was mixed in the same tube and inject i. p. in simultaneously. Mannan only and unfiltered or filtered curdlan was included as positive, respectively negative control in both settings. The graphs are based on one experiment and includes mice that have been housed in closed cages. Statistics were done with Mann-Whitney T test. Mean±SEM. *P<0.05, **P<0.01, ***P<0.001 and were the color indicates which mannan-curdlan combination it is significant against.

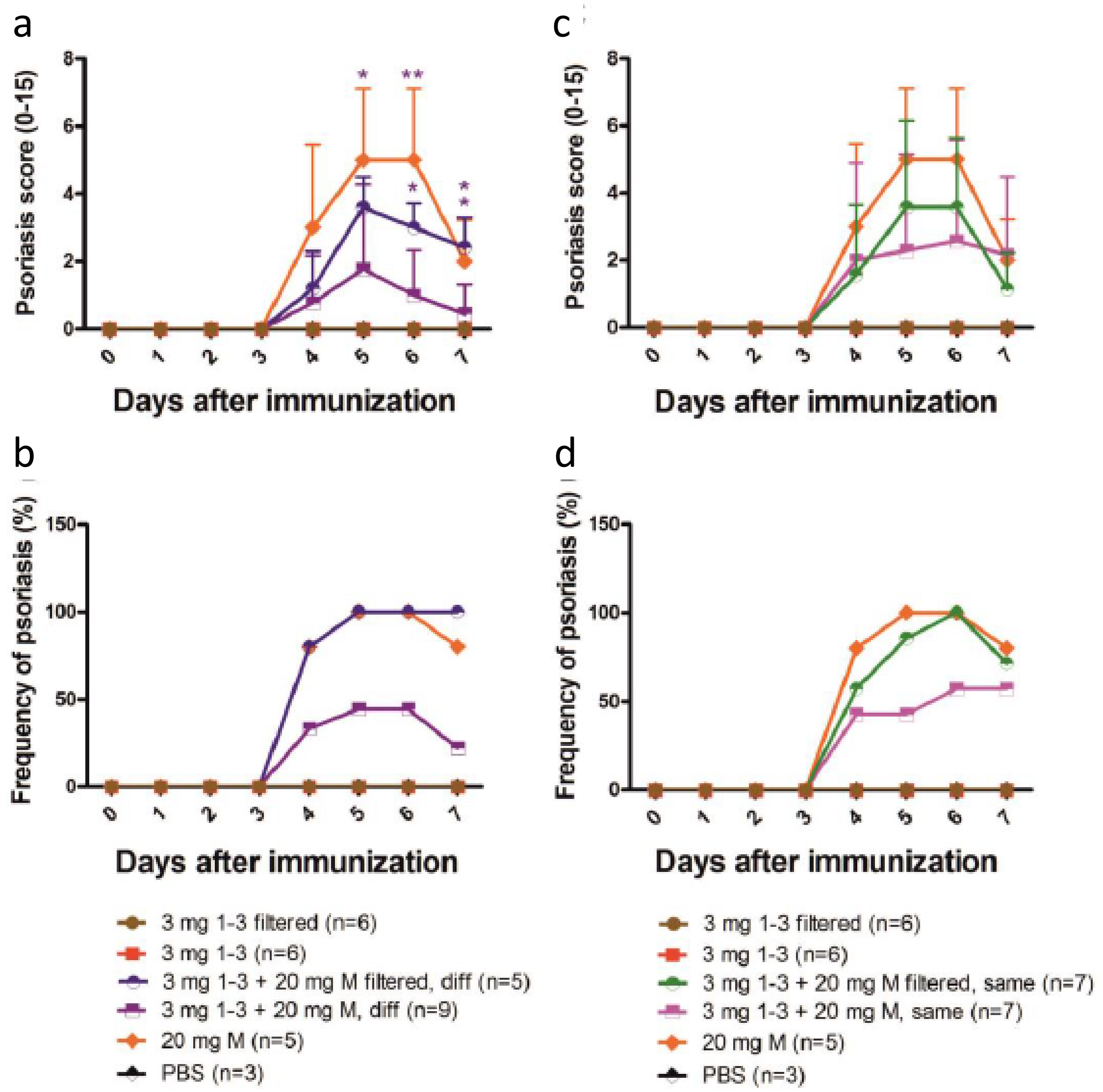

Supplementary Figure 2. Comparison of filtered and unfiltered glucan upon psoriatic lesions. a-b) A suspension of curdlan (1,3-β-glucan) used unfiltered (orange squares) and as filtered (brown circles) cannot induce psoriasis. In parallel mice were treated with mannan alone (orange diamond) or in combination with filtered (blue half-filled triangles) or unfiltered curdlan (purple half-filled squares). Mannan and curdlan was injected at different sides of the peritoneum. c-d) As a comparison (in parallel with a-b), non-filtered (light purple half-filled square) and filtered curdlan (green half-filled circle) in combination with mannan as a control was inject i. p. in the mice simultaneously. Mannan only and unfiltered or filtered curdlan was included as positive, respectively negative control in both settings. The graphs are based on one experiment and includes mice that have been housed in closed cages. Statistics were done with Mann-Whitney U test, where mean is ±SEM. *P<0.05, **P<0.01 and were the color indicates which mannan-curdlan combination it is significant against.

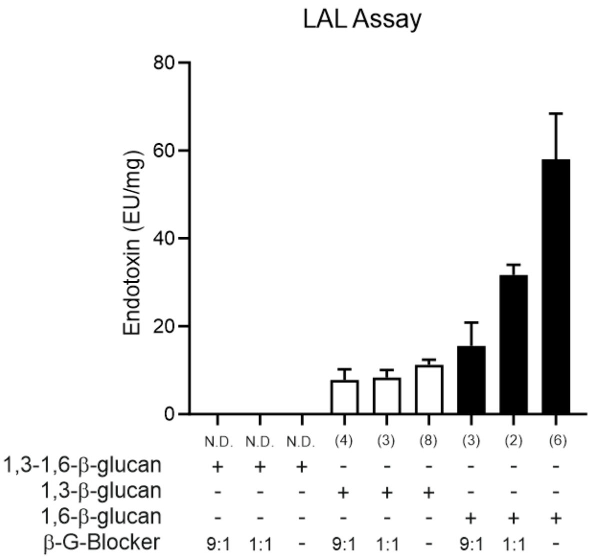

Supplementary Figure 3. Endotoxin contamination in the polysaccharides 1,3-1,6-β-glucan (Glucan from S. cerevisiae; serial dilutions up to 5 mg/ml), 1,3-β-glucan (Curdlan; up to 3 mg/ml) and 1,6-β-glucan (Pustulan; up to 3 mg/ml) was determined with the LAL assay in the presence or in the absence of β-G-Blocker (Vol (β-G-Blocker):Vol (glucan) 1:1 or 9:1). Number of the observations for which the endotoxin concentration could be determined from the standard curve are shown in parentheses. Data is presented as mean ±SEM. N.D. = not detected.

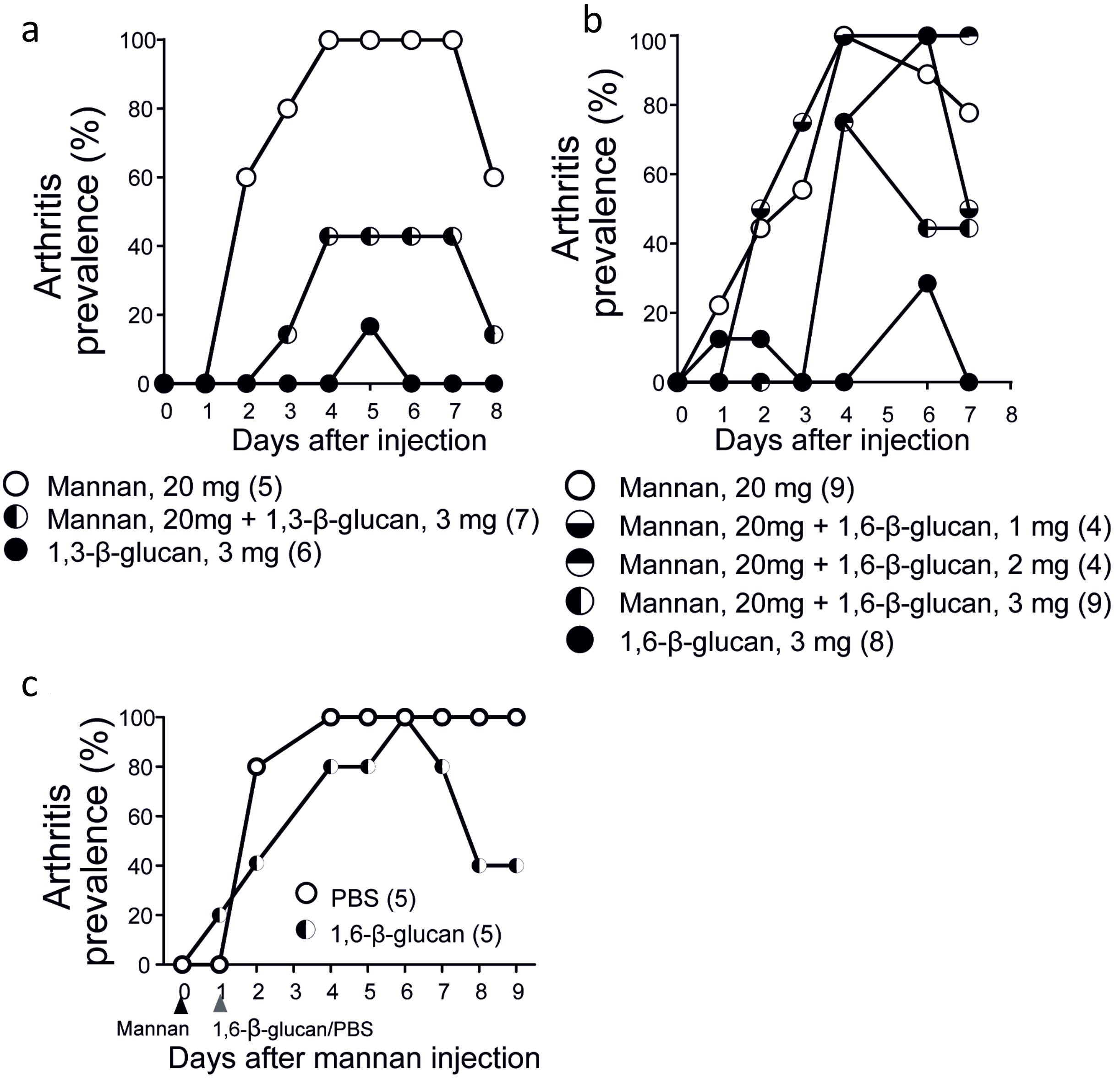

Supplementary Figure 4. The effect of β-glucan on arthritis prevalence was investigated when MIP was induced in *Ncf1* deficient mice with or without co-administration of 1,3-β-glucan (a) or 1,6-β-glucan (b), and when MIP was induced in *Ncf1* deficient mice on day 0, and 3mg 1,6-β-glucan administered one day later (c). Number of mice in each group is shown in parentheses. The data in (b) is pooled from two experiments, except the doses of 1 mg and 2 mg which were included only in one experiment.

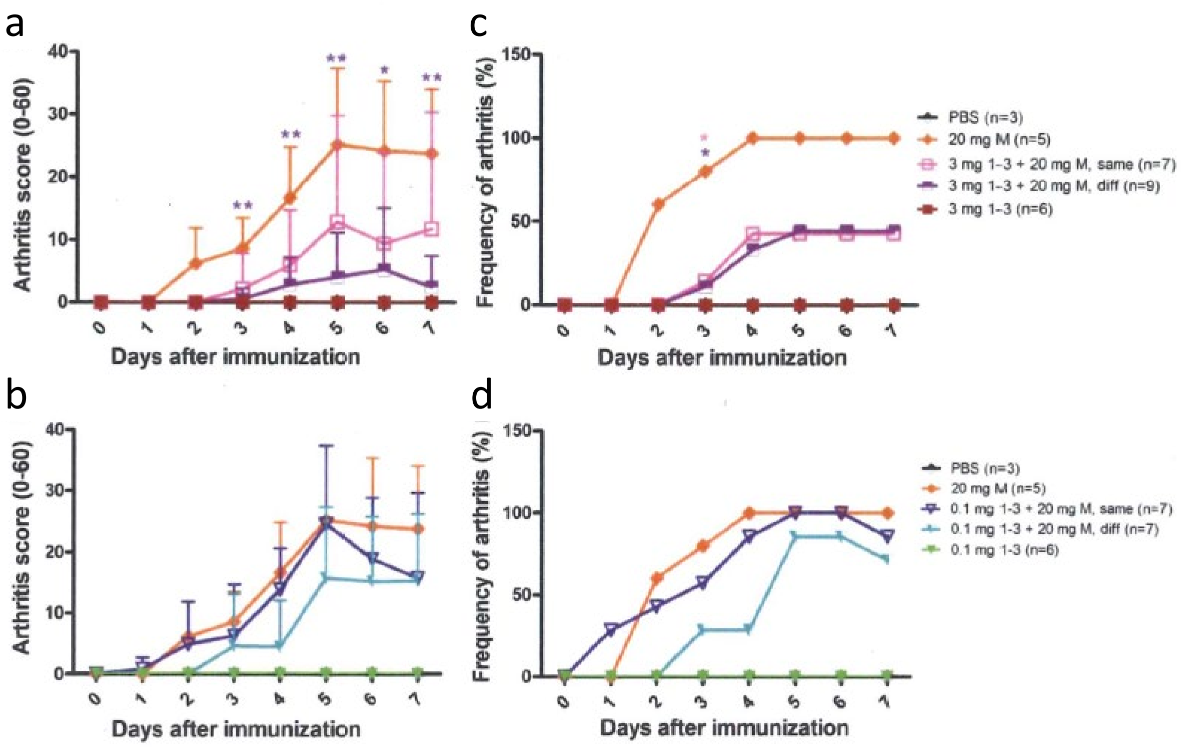

Supplementary Figure 5. 0.1mg of β-glucan is insufficient to protect against arthritic disease a-b) A suspension of 3mg curdlan (1,3-β-glucan) injected either at the same side of the peritoneum (open squares) or different sides of the peritoneum (purple squares) compared to 20mg mannan. As a positive respectively negative control, mice were treated with mannan alone (orange) or glucan alone (brown squares). c-d In a parallel experiment a dose of 0.1mg curdlan was used in combination with mannan, either on same side of peritoneum (purple triangle) or different sides of the peritoneum (blue). As a positive respectively negative control, mice were treated with mannan alone (orange) or glucan alone (green). The graphs are based on one experiment and includes mice that have been housed in closed cages. Statistics were done with Mann-Whitney U test, where mean is ±SEM. *P<0.05, **P<0.01 and were the color indicates which mannan-curdlan combination it is significant against.

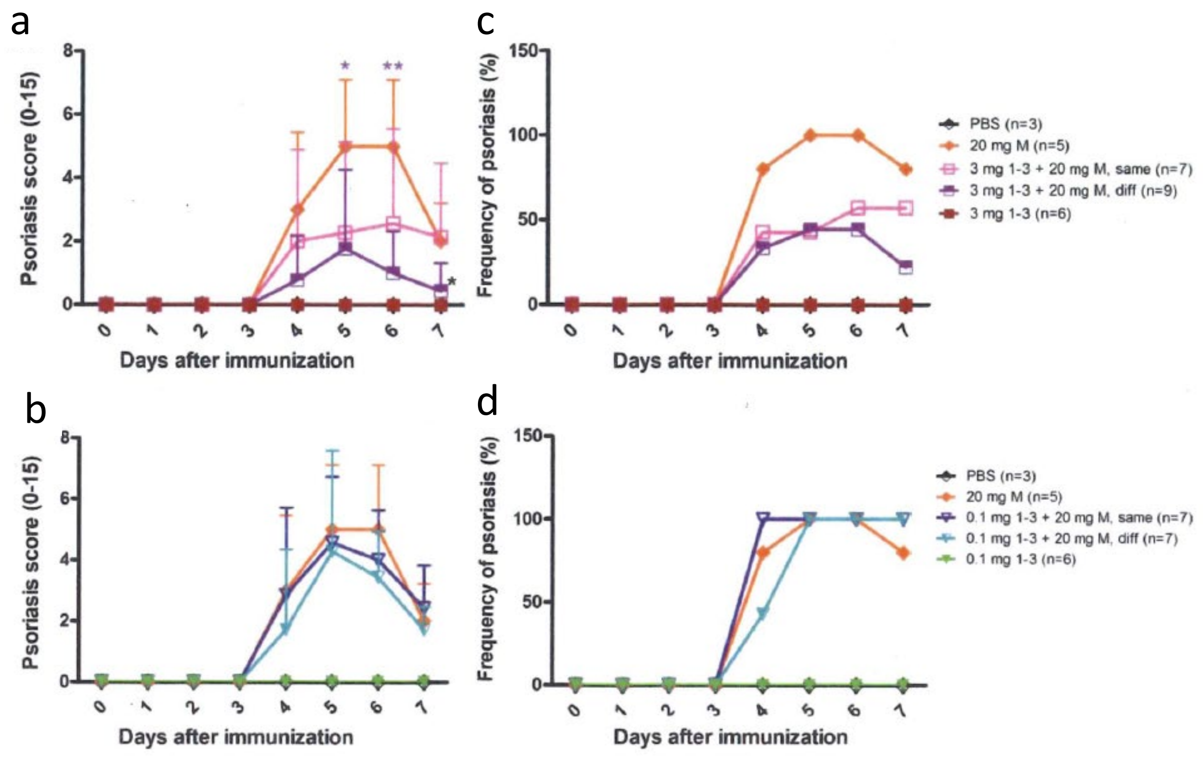

Supplementary Figure 6. 0.1mg of β-glucan is insufficient to protect against psoriatic lesions a-b) A suspension of 3mg curdlan (1,3-β-glucan) injected either at the same side of the peritoneum (open squares) or different sides of the peritoneum (purple squares) compared to 20mg mannan. As a positive respectively negative control, mice were treated with mannan alone (orange) or glucan alone (brown squares). c-d) In a parallel experiment a dose of 0.1mg curdlan was used in combination with mannan, either on same side of peritoneum (purple triangle) or different sides of the peritoneum (blue). As a positive respectively negative control, mice were treated with mannan alone (orange) or glucan alone (green). The graphs are based on one experiment and includes mice that have been housed in closed cages. Statistics were done with Mann-Whitney U test, where mean is ±SEM. *P<0.05, **P<0.01 and were the color indicates which mannan-curdlan combination it is significant against.

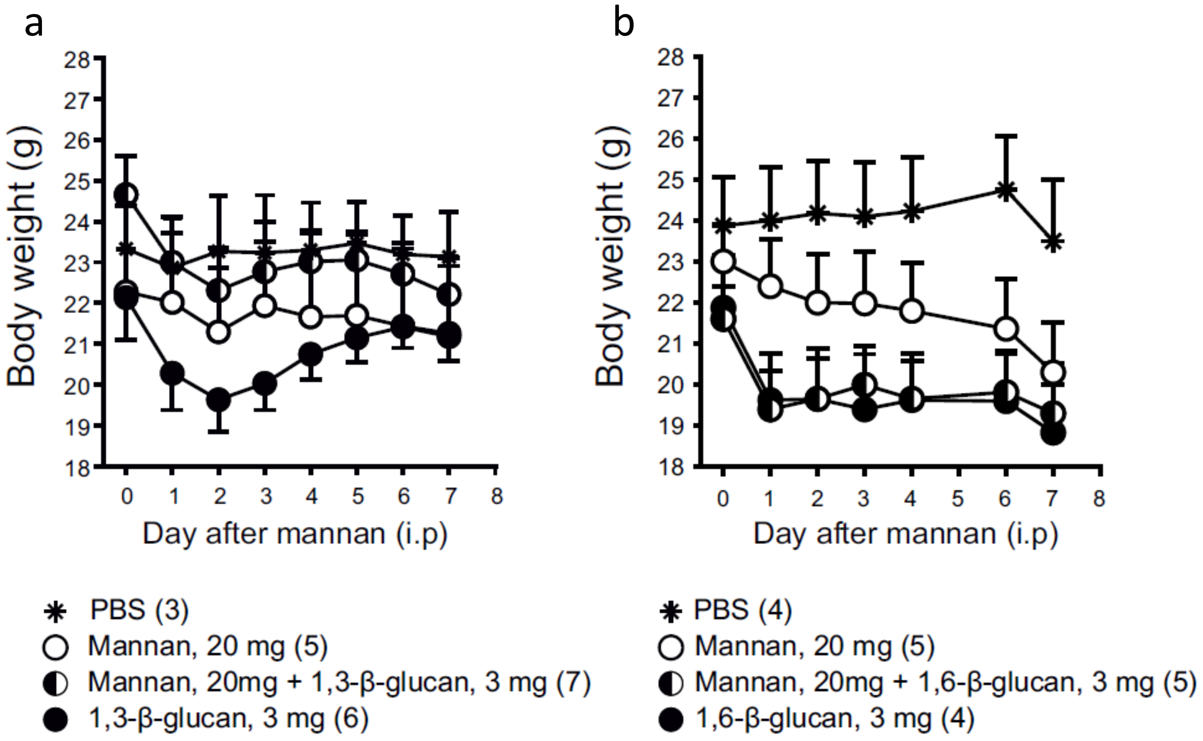

Supplementary Figure 7. The effect of β-glucan on mouse body weight was investigated when MIP was induced in *Ncf1* deficient mice with or without co-administration of 1,3-β-glucan (a) or 1,6-β-glucan (b). Number of mice in each group is shown in parentheses. Results are presented as mean with SEM.

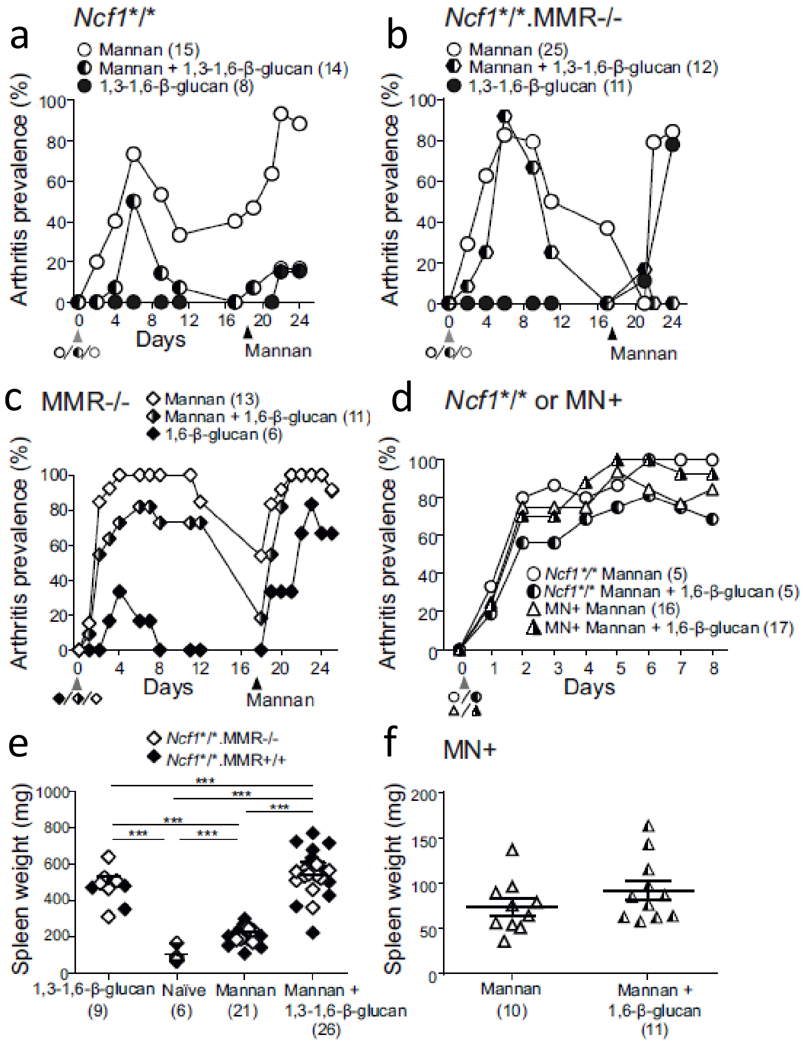

Supplementary Figure 8. Arthritis prevalence was followed when MIP was induced in *Ncf1* deficient (a), *Ncf1* and *MMR* deficient (b) or *MMR* deficient (c) mice with or without co-administration of 1,3-1,6-β-glucan (5mg; a-b) or 1,6-β-glucan (2mg; C). After 18 days, MIP was re-induced by administration of mannan to investigate the long-term effects of β-glucans. MIP was induced in MN+ and *Ncf1* deficient mice (d) with or without co-administration of 1,6-β-glucan (2mg) to investigate the effect of *Ncf1* expressed under the human CD68 promoter on the *Ncf1* deficient background in MN+ mice. Number of mice in each group is shown in parentheses in the legends. Spleen weights at the end of the experiments (day 24 and 8, respectively) are presented in e (corresponding to experiments shown a and b) and f (corresponding to one out of two experiments in d). Statistical analyses in e-f were performed by Mann-Whitney test. Results are presented with mean and SEM. The data is from three combined experiments in a-e, from one experiment in f.

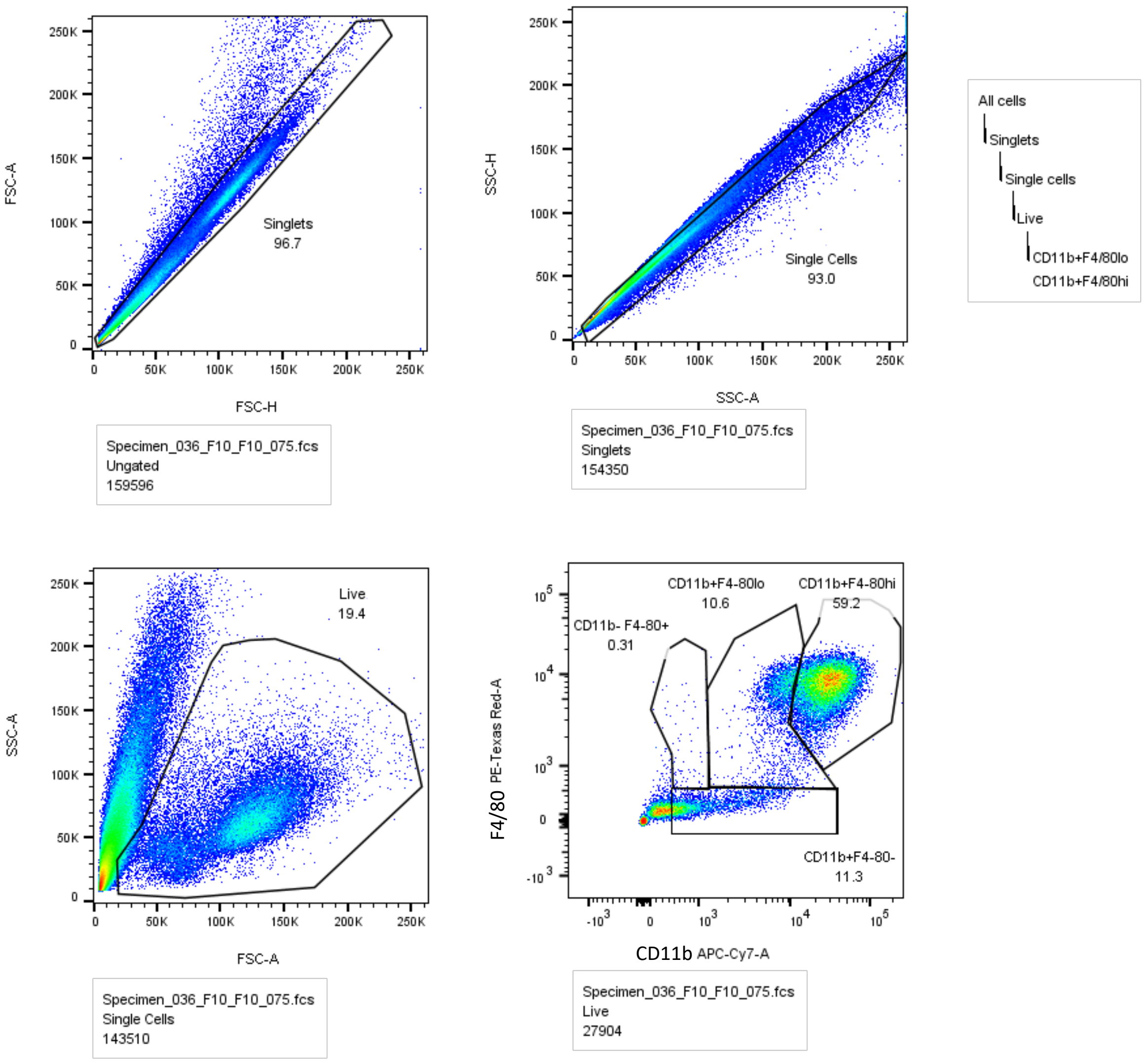

Supplementary Figure 9. Gating strategy for flow cytometry data. Doublets were firstly removed in two steps, FSC-A vs FSC-H and then SSC-A vs SSC-A. Then the live cells were gated and lastly F4/80 on PE-TexasRed and CD11b on APC-Cy7 utilized to get the target groups CD11b+F4/80lo
