# Supplementary material for: Variants of beta-glucan polysaccharides downregulate autoimmune inflammation
Source: Commun Biol. 2022 May 12;5:449. doi: 10.1038/s42003-022-03376-y (PMC9098905; doi:10.1038/s42003-022-03376-y)
Supplement: Supplementary file 3 — Reporting Summary [file 42003_2022_3376_MOESM3_ESM.pdf]

nature portfolio

Corresponding author(s): Cecilia Fahlquist Hagert, cecilia.hagert@biomed.au.dk, +46-703879996 and Rikard Holmdahl, rikard.holmdahl@ki.se, +46-(0)8-524 846 07

Last updated by author(s): Apr 21, 2022

Reporting Summary

Nature Portfolio wishes to improve the reproducibility of the work that we publish. This form provides structure for consistency and transparency in reporting. For further information on Nature Portfolio policies, see our Editorial Policies and the Editorial Policy Checklist.

Statistics

For all statistical analyses, confirm that the following items are present in the figure legend, table legend, main text, or Methods section.

n/a	Confirmed	
☐	☒	The exact sample size (n) for each experimental group/condition, given as a discrete number and unit of measurement
☐	☒	A statement on whether measurements were taken from distinct samples or whether the same sample was measured repeatedly
☐	☒	The statistical test(s) used AND whether they are one- or two-sided<br>*Only common tests should be described solely by name; describe more complex techniques in the Methods section.*
☐	☒	A description of all covariates tested
☒	☐	A description of any assumptions or corrections, such as tests of normality and adjustment for multiple comparisons
☐	☒	A full description of the statistical parameters including central tendency (e.g. means) or other basic estimates (e.g. regression coefficient) AND variation (e.g. standard deviation) or associated estimates of uncertainty (e.g. confidence intervals)
☒	☐	For null hypothesis testing, the test statistic (e.g. F, t, r) with confidence intervals, effect sizes, degrees of freedom and P value noted<br>*Give P values as exact values whenever suitable.*
☒	☐	For Bayesian analysis, information on the choice of priors and Markov chain Monte Carlo settings
☒	☐	For hierarchical and complex designs, identification of the appropriate level for tests and full reporting of outcomes
☒	☐	Estimates of effect sizes (e.g. Cohen's d, Pearson's r), indicating how they were calculated

Our web collection on statistics for biologists contains articles on many of the points above.

Software and code

Policy information about availability of computer code

Data collection	n/a
Data analysis	GraphPad Prism, version 5 for making the graphs and statistics and FlowJO software V10 (Tree Star) for analyzing flow cytometry data

For manuscripts utilizing custom algorithms or software that are central to the research but not yet described in published literature, software must be made available to editors and reviewers. We strongly encourage code deposition in a community repository (e.g. GitHub). See the Nature Portfolio guidelines for submitting code & software for further information.

Data

Policy information about availability of data

All manuscripts must include a data availability statement. This statement should provide the following information, where applicable:

- Accession codes, unique identifiers, or web links for publicly available datasets
- A description of any restrictions on data availability
- For clinical datasets or third party data, please ensure that the statement adheres to our policy

The data that support the findings of this study are available from the corresponding authors upon reasonable request

Field-specific reporting

Please select the one below that is the best fit for your research. If you are not sure, read the appropriate sections before making your selection.

☒ Life sciences ☐ Behavioural & social sciences ☐ Ecological, evolutionary & environmental sciences

For a reference copy of the document with all sections, see nature.com/documents/nr-reporting-summary-flat.pdf

Life sciences study design

All studies must disclose on these points even when the disclosure is negative.

Sample size	Size of the experiments were a minimum of three for each group and replication and a the maximum in each group was determined upon litter size. Following the 3Rs.
Data exclusions	Exclusion was only done if the mice were suffering severe weight-loss, or exhibited other signs of poor well being.
Replication	All experiments were successfully replicated and all replications are included in the graphs.
Randomization	The mice were randomized in their groups.
Blinding	During macroscopic scoring the investigator were blinded for the groups and the grouping reveal after end of experiment. Mice from different groups were mixed in their cages.

Reporting for specific materials, systems and methods

We require information from authors about some types of materials, experimental systems and methods used in many studies. Here, indicate whether each material, system or method listed is relevant to your study. If you are not sure if a list item applies to your research, read the appropriate section before selecting a response.

Materials & experimental systems

n/a	Involved in the study	
☐	☒	Antibodies
☒	☐	Eukaryotic cell lines
☒	☐	Palaeontology and archaeology
☐	☒	Animals and other organisms
☒	☐	Human research participants
☒	☐	Clinical data
☒	☐	Dual use research of concern

Methods

n/a	Involved in the study	
☒	☐	ChIP-seq
☐	☒	Flow cytometry
☒	☐	MRI-based neuroimaging

Antibodies

Antibodies used	Please see Method section
Validation	All flow cytometry experiments were run with FMO controls. In the initial experiment and where needed for analyzation, a well with a single antibody stain was included.

Animals and other organisms

Policy information about studies involving animals; ARRIVE guidelines recommended for reporting animal research

Laboratory animals	BQ.Ncf1m1J mice (denoted here as Ncf1*/*) and MN+ mice with an Ncf1m1J mutation, but expressing WT Ncf1 under human CD68 promoter. CD206 (macrophage mannose receptor, MMR) deficient mice. The mice have been back-crossed more than 10 times to BQ background and were used here with (Ncf1*/*.MMR-/-) or without (MMR-/-) the Ncf1m1J mutation.
Wild animals	n/a
Field-collected samples	n/a
Ethics oversight	All experiments were carried out under ethical permit numbers ESAVI-0000497/041003/2011 and ESAVI/439/04.10.07/2017 at the University of Turku and the Karolinska Institute under the permit number N490/12 and N35/16.

Note that full information on the approval of the study protocol must also be provided in the manuscript.

Flow Cytometry

Plots

Confirm that:

☒ The axis labels state the marker and fluorochrome used (e.g. CD4-FITC).

☒ The axis scales are clearly visible. Include numbers along axes only for bottom left plot of group (a 'group' is an analysis of identical markers).

☒ All plots are contour plots with outliers or pseudocolor plots.

☒ A numerical value for number of cells or percentage (with statistics) is provided.

Methodology

Sample preparation	Peritoneal cells were collected by washing the peritoneum using PBS injected with a syringe and cells were there after counted and equally plated for staining.
Instrument	BD Fortessa (BD Biosciences)
Software	FlowJO software V10 (Tree Star).
Cell population abundance	The cells were not sorted
Gating strategy	The boundaries were determined in accordance with the FMO controls.

☒ Tick this box to confirm that a figure exemplifying the gating strategy is provided in the Supplementary Information.
